# The Study on the Regulation of Th Cells by Mesenchymal Stem Cells Through the JAK-STAT Signaling Pathway to Protect Naturally Aged Sepsis Model Rats

**DOI:** 10.3389/fimmu.2022.820685

**Published:** 2022-02-07

**Authors:** Lu Wang, Zihui Deng, Yan Sun, Yan Zhao, Yun Li, Mengmeng Yang, Rui Yuan, Yuyan Liu, Zhirong Qian, Feihu Zhou, Hongjun Kang

**Affiliations:** ^1^Medical School of Chinese People’s Liberation Army (PLA), Beijing, China; ^2^Department of Critical Care Medicine, the First Medical Center, Chinese People’s Liberation Army (PLA) General Hospital, Beijing, China; ^3^Department of Basic Medicine, Graduate School, Chinese People’s Liberation Army (PLA) General Hospital, Beijing, China; ^4^School of Public Health, Capital Medical University, Beijing, China; ^5^Scientific Research Center, The Seventh Affiliated Hospital, Sun Yat-sen University, Shenzhen, China

**Keywords:** sepsis, mesenchymal stem cells, immune function, inflammatory response, helper T cells

## Abstract

Sepsis is the leading cause of death among patients, especially elderly patients, in intensive care units worldwide. In this study, we established a sepsis model using naturally aged rats and injected 5×10^6^ umbilical cord-derived MSCs *via* the tail vein. Each group of rats was analyzed for survival, examined for biochemical parameters, stained for organ histology, and analyzed for the Th cell subpopulation ratio and inflammatory cytokine levels by flow cytometry. Western blotting was performed to detect the activity of the JAK-STAT signaling pathway. We designed the vitro experiments to confirm the regulatory role of MSCs, and verified the possible mechanism using JAK/STAT inhibitors. It was revealed from the experiments that the 72 h survival rate of sepsis rats treated with MSCs was significantly increased, organ damage and inflammatory infiltration were reduced, the levels of organ damage indicators were decreased, the ratios of Th1/Th2 and Th17/Treg in peripheral blood and spleen were significantly decreased, the levels of pro-inflammatory cytokines such as IL-6 were decreased, the levels of anti-inflammatory cytokines such as IL-10 were increased, and the levels of STAT1 and STAT3 phosphorylation were reduced. These results were validated in *in vitro* experiments. Therefore, this study confirms that MSCs can control the inflammatory response induced by sepsis by regulating Th cells and inflammatory factors, and that this leads to the reduction of tissue damage, protection of organ functions and ultimately the improvement of survival in aged sepsis model rats. Inhibition of the JAK-STAT signaling pathway was surmised that it may be an important mechanism for their action.

## Introduction

Sepsis is a life-threatening organ dysfunction caused by a dysregulated host response to infection (1) and is the leading cause of death for intensive care unit (ICU) patients, especially among the elderly. Globally, there are 30 million cases of sepsis each year with a mortality rate of 30-40% (2, 3), and more than 60% of the deceased are elderly people over 65 years of age (4). In recent years, a combination of anti-infective, anti-shock and supportive therapy has reduced the high mortality from sepsis, but the severity of it has increased year by year (5). Recent studies have found that the immune system plays a central role in curing sepsis both in the early storm of inflammatory factors and in the later stages of immune suppression (6, 7). The incidence of sepsis will also continue to increase along with the increasing aging of population (8).

Mesenchymal stem cells (MSCs) interact with many types of immune cells and regulate their functions, such as metabolism, migration, cytotoxicity and inflammatory response (9). Therefore, MSCs can be used to treat a variety of autoimmune diseases, inflammation-related diseases, and other diseases triggered by the dyshomeostasis in immune system. Currently, MSCs have been used by researchers worldwide for the treatment of sepsis. In animal experiments, MSCs have been found to reduce the systemic inflammatory response, decrease the level of organ damage (10), and improve the survival of sepsis model animals (11).

The JAK/STAT pathway is considered as one of the major signaling pathways involved in sepsis (12) and is a part of many key cytokine signaling pathways in the pathogenesis of sepsis, such as interleukin (IL)-4, IL-6, IL-10, IL-12, and interferon (IFN)-γ (13–15). In the early onset of sepsis, the body responds to infection with strong immune responses, that are manifested by the massive mobilization of pro-inflammatory cells and pro-inflammatory factors (16), which significantly elevates the levels of pro-inflammatory cytokines such as IL-1β and IL-6, and causes an imbalance in the helper T cell (Th) 1/Th2 and Th17/Treg ratios (17–19). A prospective observational study found that the imbalanced ratio in the Th17/Treg was correlated with the severity and prognosis of sepsis patients (20). The inflammatory factor storm in the early stages of sepsis would cause massive depletion of lymphocytes and leads to the long-term immune dysfunction (21), increased susceptibility to secondary infections, and reduced the 5-year survival rate (22, 23). Currently, MSCs are widely used in the treatment of autoimmune diseases and acute/chronic inflammation, and a large number of studies have found that MSCs can regulate the helper T cell subsets represented by Th1 and Th17 cells.

However, the experimental animals investigated in the current studies were all adult laboratory animals and to our knowledge, no studies have reported on the application of MSCs to naturally aged animals, which is inconsistent with the clinical situation that the majority of sepsis patients are the elderly. In this study, we established a sepsis model by the cecal ligation and puncture (CLP) method used on naturally aged Sprague Dawley (SD) rats, observed the effect of MSCs on improving survival and vital organ functions in aged sepsis model rats, clarified the inhibitory effect of MSCs on the early inflammatory factor storm and the regulatory effect of helper T cell subsets in sepsis of the aged experimental objects and explored the molecular mechanisms of such effects.

## Material and Methods

### Study Design

This study was approved by the Ethics Committee of the Chinese PLA General Hospital. Animal experiments were approved by the Experimental Animal Welfare Ethics Committee of Chinese PLA General Hospital. The selected experimental animals were ninety (90) 21-month-old male SD rats (SPF (Beijing) Biotechnology, Beijing, China) weighing 801.5 ± 79.5 g. The rats were divided into sham-operated group (n=18), sepsis model group (n=36) and sepsis+MSCs treatment group (n=36). In each group, rats were randomly and equally assigned to 3 subgroups and samples were collected after euthanasia at 6 h, 24 h or 72 h post-operation and/or treatment. Not all rats were included in the analysis because of mortality in both the model and treatment group rats during the experiment. The CLP model was used as the model of sepsis, and umbilical cord-derived mesenchymal stem cells (UC-MSCs) were injected into the tail vein 1 h after CLP operation to cure sepsis. Five million (5×10^6^) UC-MSCs in 1 ml saline were applied to each animal in the treatment group, and the rats in sham-operated and model groups were injected with equal amounts of sterile saline at the same time. The 72 h survival rate of the rats in each group was statistically analyzed.

The vitro experiments were performed by using lymphocytes isolated from the spleen of adult SD rats. Cells were either left unstimulated (Control Group), stimulated with LPS (Model Group), or stimulated with LPS and co-cultured with MSC (Treatment Group). This part of the experiment was analyzed for lymphocytes and supernates at 6h, 24h and 72h, respectively. In addition, another part of the *in vitro* experiment was performed to validate the regulation of the JAK-STAT signaling pathway by MSCs using the JAK inhibitor AZD1480 (5μM, Cat# M2044, AbMole, TX, USA), the STAT1 inhibitor fludarabine (100μM, Cat# M2028, AbMole, TX, USA), and the STAT3 inhibitor cryptotanshinone (50μM, Cat# M3982, AbMole, TX, USA).

### Cell Culture

Umbilical cords were obtained from the Department of Obstetrics and Gynecology, the First Medical Center, Chinese PLA General Hospital, China, and consent was obtained from the parturients. Umbilical cord-derived MSCs were isolated, cultured, and expanded under the scheme from the previously published study (24). In brief, the UC-MSCs were cultured in the MEM medium (Gibco, Thermo Fisher Science, Cat# 12571063, MA, USA) supplemented with 10% fetal bovine serum (Gibco, Thermo Fisher Science, Cat# 10099141, MA, USA). When cells reached to 70-80% confluence, they were detached with trypsin-ethylenediaminetetraacetic acid (Gibco, Cat# 25300054) and passaged. SD rat spleen lymphocytes were cultured in RPMI 1640 medium (Gibco, Thermo Fisher Science, Cat# 31870082, MA, USA) with 10% fetal bovine serum (Gibco, Thermo Fisher Science, Cat# 10099141, MA, USA).

### Cecal Ligation and Perforation (CLP)

A sepsis model was established by the modified CLP method (25). The rats were anesthetized by intraperitoneal injection of 50 mg/kg sodium pentobarbital to ensure that the rats would not wake up until the surgery was completed. After each rat was anesthetized with the abdominal body hair removed and the skin was disinfected, a 1.5-cm-long midline incision was made on the abdomen, the cecum was located and gently externalized, then it was ligated at 1/2 of its length with a sterile 4-gauge wire, and a small amount of intestinal contents was extruded after penetrating the cecum once at the distal end with a 22G needle. Finally, the cecum was placed back into the abdominal cavity, and the abdominal muscles and skin were sutured layer by layer. The rats in the sham-operated group only underwent open cecum externalization without ligation or perforation.

### Identification of Umbilical Cord-Derived Mesenchymal Stem Cells

Umbilical cord MSCs were identified as previously described (26). Third passage (P3) MSCs were immunophenotyped by flow cytometry. Cells were collected and adjusted to 1× 10^6^ cells per sample and were washed with PBS and incubated with antibodies for 15 min at room temperature protected from light. Following antibodies were obtained from eBioScience, Thermo Fisher Science (MA, USA): PE CD11b monoclonal antibody (Cat# RM2804), PE CD34 monoclonal antibody (Cat# 12-0349-41), APC CD44 monoclonal antibody (Cat#47-0441-82), APC CD45 monoclonal antibody (Cat#4 7-0441-82), APC CD45 monoclonal antibody (Cat# 47-0451-82), APC CD90 monoclonal antibody (Cat# 17-0909-41), and PE CD105 monoclonal antibody (Cat# MA5-17946). After incubation, the cells were washed with PBS and analyzed by flow cytometry using the BD Accuri C6 software system (version 1.0.264.21; BD Biosciences). Differentiation potential was analyzed by using human mesenchymal stem cell differentiation kits (TBDscience, Tianjin, China). P3 MSCs were cultured in the 6-well culture plates at a density of 10^4^ cells/well, and lipogenic (Cat# TBD20190004), osteogenic (Cat# TBD20190002) and chondrogenic (Cat# TBD20190003) differentiation assays were performed and evaluated according to the kit instructions. Cell identification results are shown in **Supplementary Material 1**.

### Histology and Tissue Staining

The right lung, liver, kidney, ileum and spleen of each rat from the experimental group were collected for histological observation and staining. The organs were fixed in 4% paraformaldehyde, dehydrated and paraffin embedded. Paraffin sections were analyzed by hematoxylin-eosin (HE) staining (Cat# G1120, Solarbio, Beijing, China) to examine gross histology. TdT-mediated dUTP nick-end labeling (TUNEL) staining (Cat# C1088, Beyotime, Shanghai, China) was used to label apoptotic cells in the rat spleen. The stained sections were visualized and scanned with the panoramic MIDI CaseViewer system (3DHISTECH, Hungary).

### Biochemical Assays

Blood samples from each experimental group of rats were centrifuged at 2000 x g for 10 min within 4 h of collection to isolate the supernate. Alanine aminotransferase (ALT), aspartate aminotransferase (AST), alkaline phosphatase (ALP), lactate dehydrogenase (LDH), creatinine (CREA) and uric acid (UA) were measured by an automated biochemical analyzer (7170-A, Hitachi, Japan). Immunocytokine concentrations in the serum and spleen tissue homogenates were measured using the Aimplex Multiple Immunoassays for Flow on a flow cytometer (BD Bioscience, NJ, USA) according to the manufacturer’s (Aimplex Bioscience, CA, USA) instructions, including IL-1β (Cat# B311165), IL-6 (Cat# A311125), IL-17A (Cat# B311113), TNF-α (Cat# A311129), IFN-γ (Cat# A311101), IL-4 (Cat# A311121), IL-10 (Cat# A311109), IL-13 (Cat# B311177) and TGF-β (Cat# B111206) were measured.

### Flow Cytometry

Flow cytometry (BD Bioscience, NJ, USA) was used to analyze the helper T cell subsets in peripheral blood and spleen cells suspensions according to the reagent manufacturer’s (Biolegend, CA, USA) instructions. Peripheral blood was collected into containers with sodium heparin at a concentration of 1000 U/ml as anticoagulant, and spleen tissue was grounded and treated similarly as described before. We used FITC anti-rat CD4 (Cat# 201505), anti-rat CD45 PerCP-CY5.5 (Cat# 202220) to label the helper T cells; APC anti-rat CD3 (Cat# 201414) and PE anti-mouse/rat/human FOXP3 (Cat# 320008) were adopted to label Treg cells; other labels are Alexa Fluor^®^ 647 anti-rat IFN-γ (Cat# 507810) for Th1 cells, PE anti-rat IL-4 (Cat# 511906) for Th2 cells, and Alexa Fluor^®^ 647 anti-mouse IL-17 (Cat# 146303) for Th17 cells. The aforementioned four cells were stained intracellularly by using Foxp3/transcription factor fixation/permeabilization concentrates and dilutions (Cat# 00-5521-00, eBioScience, Thermo Fisher Science, MA, USA). Th1, Th2 and Th17 cells were assayed after 5 h of stimulation with Cell Stimulation Cocktail (Cat# TNB-4975-UL100, Tonbo Bioscience, CA, USA). Fixation, stimulation and staining were performed according to the manufacturers’ instructions and analyzed by flow cytometry. The results of flow cytometry presented that CD45^+^ cell populations were considered as leukocytes, where CD4^+^ cells were considered as helper T lymphocytes, and then Th1 (IFN-γ^+^), Th2 (IL-4^+^), Th17 (IL-17A^+^) and Treg (CD25^+^FoxP3^+^) were analyzed by their respective specific antibodies.

### Western Blot

The spleen was homogenized in a low-temperature high-speed grinder (Servicebio, Wuhan, China) to obtain the homogenate. Lymphocytes for the vitro experiments were collected at pre-determined time points, rinsed three times with pre-cooled PBS (Servicebio, Wuhan, China), and split on ice for 10 min with RIPA lysis buffer (Thermo Fisher Scientific, MA, USA). BCA quantification kit (Solarbio, Beijing, China) was used for the sample protein quantification, followed by the incubation in a metal bath at 95°C for 10 min. Protein samples were transferred onto nitrocellulose membranes after sodium dodecyl sulfate-polyacrylamide gel electrophoresis (SDS-PAGE) at 100 V. The membranes were blocked with Fast Block (NcmBlot Blocking Buffer) (Cat# P30500, New Cell & Molecular Biotech, Suzhou, China) for 10 min and incubated with primary rabbit polyclonal antibody overnight at 4°C. Next day, the membranes were incubated with horseradish peroxidase (HRP)-linked goat anti-rabbit IgG (Cat# 7074S, 1:3000, CST, MA, USA) at room temperature for 120 min, and washed with 1× TBST (Solarbio, Beijing, China). Protein bands were viewed and documented using an electrochemiluminescence kit (Solarbio, Beijing, China). Primary antibodies used in this study included STAT1 (Cat# 14994, 1:1000, CST), p-STAT1 (Cat# 7649, 1:1000, CST), STAT3 (Cat# 12640, 1:1000, CST), p-STAT3 (Cat# 9145, 1:2000, CST) and β-Actin (Cat# 8457, 1:2000, CST).

### Statistical Analysis

SPSS 22.0 statistical software was used for analysis where the measurement data were expressed as x ± s. When the data of each group conformed to normal distribution (Kolmogorov-Smirnov test) and chi-square (Levene test), one-way ANOVA was used for comparison of multiple sample means. A nonparametric test (Mann-Whitney U test) was used for pathological damage scores, Kaplan-Meier method was used for survival analysis of all rats, and the statistical method applied was the log-rank (Mantel-Cox) test. p < 0.05 was considered a statistically significant difference in this study and the graphs were plotted by GraphPad Prism 8.0.

## Results

### Improvement of Survival and Biochemical Parameters of MSCs Treatment in Aged Sepsis Rats

**Figure 1:**
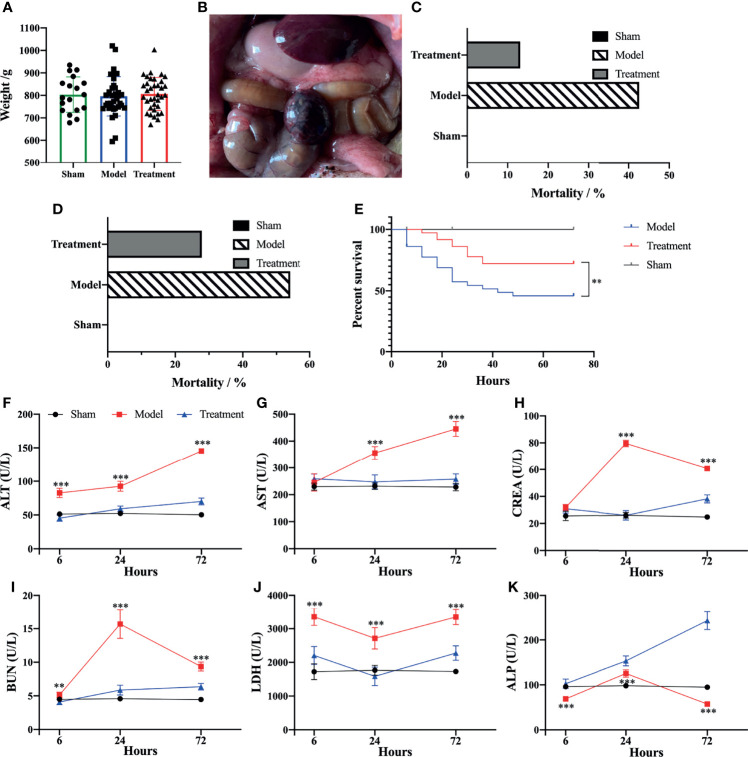
Survival rate and biochemical markers of rats in each group. **(A)** Mean body weight of rats in each group; **(B)** Hemorrhagic necrosis of cecum and abdominal bulk of rats after CLP; **(C)** 24h mortality rate of rats in each group; **(D)** 72h mortality rate of rats in each group; **(E)** survival analysis of rats in each group [Log-rank (Mantel-Cox) Test]; **(F–K)** levels of biochemical markers in serum of rats in each group. n=5, **p<0.01, ***p<0.001.

It was found that the average body weight of the rats was not significantly different among each group and met the weight criteria for aged rats (**Figure 1A**). A sepsis model was successfully established by the CLP method (**Figure 1B**). UC-MSCs possessed lipogenic, osteogenic and chondrogenic differentiation abilities, and the expression of surface markers was accord with the related criteria (26) (**Supplementary Figure 1**). The mortality rate of aged sepsis model rats was reduced after MSC treatment (**Figures 1C, D**), and the 72 h survival rate in the treatment group was evidently higher than that in the control-treated group (χ^2 =^ 13.56, P<0.01, **Figure 1E**). The levels of ALT, AST, CREA, BUN and LDH of aged sepsis model rats were all obviously higher than the rats in the sham-operated group. After treatment with MSCs, the levels of these biochemical indices became distinctly lower while the levels of ALP became significantly higher. These results indicated that hepatic and renal functions were greatly improved in aged sepsis rats after treatment with MSCs (**Figures 1F–K**).

### Amelioration on Organ Damage of MSCs Treatment in Aged Sepsis Rats

**Figure 2:**
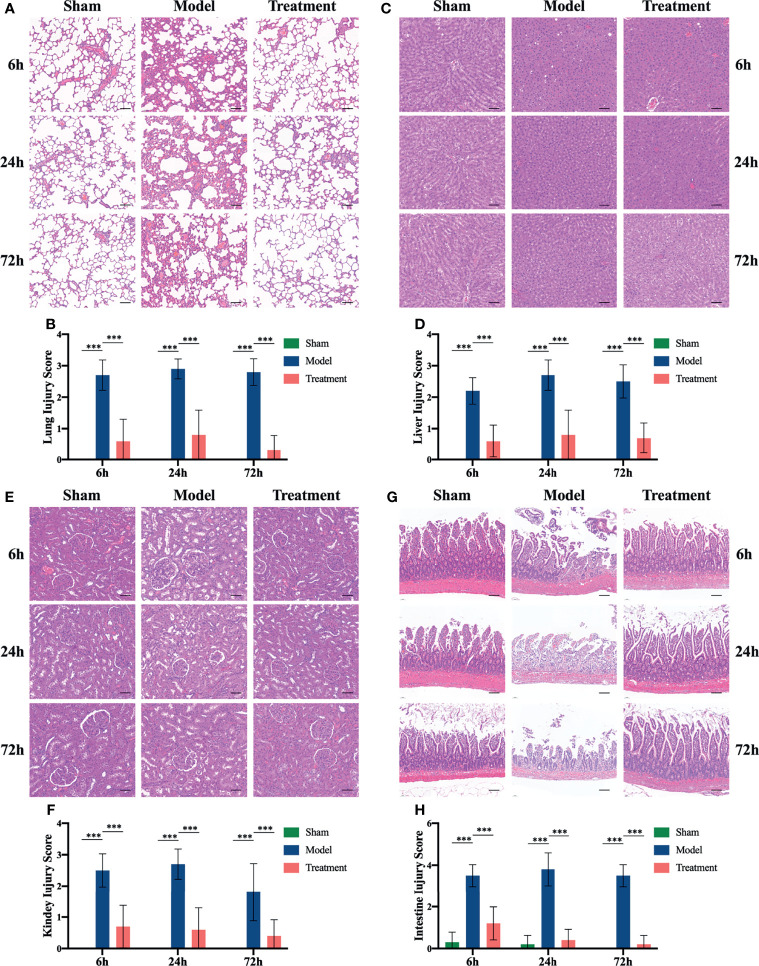
HE staining and tissue damage scores of lung, liver, kidney and ileum of rats in each group. **(A)** HE staining of lungs at 6h, 24h and 72h in each group of rats; **(B)** lung tissue damage scores in each group of rats; **(C)** liver HE staining at 6h, 24h and 72h in each group of rats; **(D)** liver tissue damage scores in each group of rats; **(E)** kidney HE staining at 6h, 24h and 72h in each group of rats; **(F)** kidney tissue damage scores in each group of rats; **(G)** ileum HE staining at 6h, 24h and 72h in each group of rats; **(H)** ileal tissue damage scores of rats in each group. n=5, scale bar=100μm, ***p<0.001.

MSCs could reduce the histopathological damage to critical organs, including the lung, liver, kidney and intestine. The alveolar structure of rats in the control-treated group were disordered and the septa was widened; a large number of inflammatory cell infiltrates were observed. In contrast, rats in the treatment group showed obvious less lung damage that the tissue damage scores were lower than the control-treated group (**Figures 2A, B**). The control-treated group rats showed massive inflammatory cell infiltration in the liver at the early onset of the sepsis, which was followed by hepatocellular edema, structural disorder of liver lobules, vacuolar degeneration and piecemeal necrosis; while the degree of liver injury was significantly reduced after MSCs treatment (**Figures 2C, D**). Sepsis could cause kidney injury in rats, which was manifested as interstitial edema with massive inflammatory cell infiltration and glomerulus edema. The degree of the aforementioned injury was remarkably reduced in the MSCs-treated group of rats (**Figures 2E, F**). In addition, the intestine of rats in the control-treated group was severely damaged with the evidenced showing that the epithelial layer on both sides of the villi was markedly separated from the lamina propria, the apical part was broken, and the lamina propria was heavily infiltrated with inflammatory cells, on the contrary, the intestinal damages were effectively reduced after the MSCs treatment (**Figures 2G, H**). Besides, the rats in the sham-operated group showed no significant tissue damage in these organs.

### Modulation of Systemic Inflammatory Responses of MSCs Treatment in Aged Sepsis Rats

**Figure 3:**
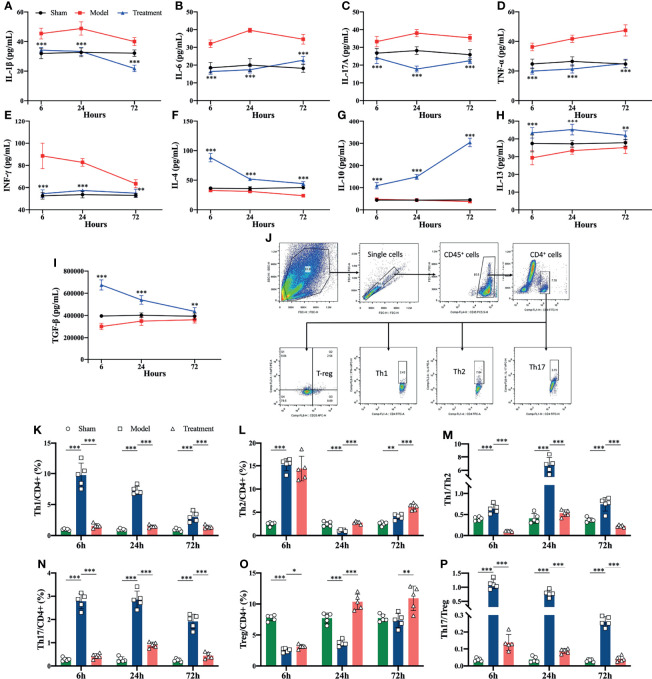
Inflammatory cytokine levels and proportions of Th cell subpopulations in peripheral blood of rats in each group. **(A–I)** Inflammatory cytokine levels in serum of rats in each group (*compared with the model group); **(J)** circle gating strategy for flow cytometry analysis of peripheral blood Th cell proportions; **(K, L, N, O)** flow analysis of the proportions of Th1, Th2, Th17, and Treg cells in peripheral blood as a percentage of CD4^+^ T cells; **(M)** ratio of Th1/Th2 in peripheral blood of rats in each group; **(P)** ratio of Th17/Treg in peripheral blood of rats in each group. n=5, *p<0.05, **p<0.01, ***p<0.001.

Pro-inflammatory cytokines (IL-1β, IL-6, IL-17A, TNF-α, IFN-γ) were elevated in the control-treated rats compared to the sham-operated rats, where the levels of IL-1β, IL-6 and IL-17A began to increase obviously in the early onset. In comparison, circulating levels of pro-inflammatory cytokines were significantly reduced in MSCs-treated rats, and anti-inflammatory cytokines (IL-4, IL 10, IL-13, TGF-β) were elevated compared to the control-treated group (**Figures 3A–I**). In addition, the proportions of Th1 and Th17 cell subsets among helper T cells rised in the peripheral blood of aged sepsis model rats, and after treatment with MSCs, the proportions of Th1 and Th17 cells were evidently decreased, and the ratios of Th1/Th2 and Th17/Treg were also distinctly reduced compared to the control-treated group (**Figures 3J–P**).

### Modulation of Splenic Cytokines and Helper T Cells of MSCs Treatment in Aged Sepsis Rats

**Figure 4:**
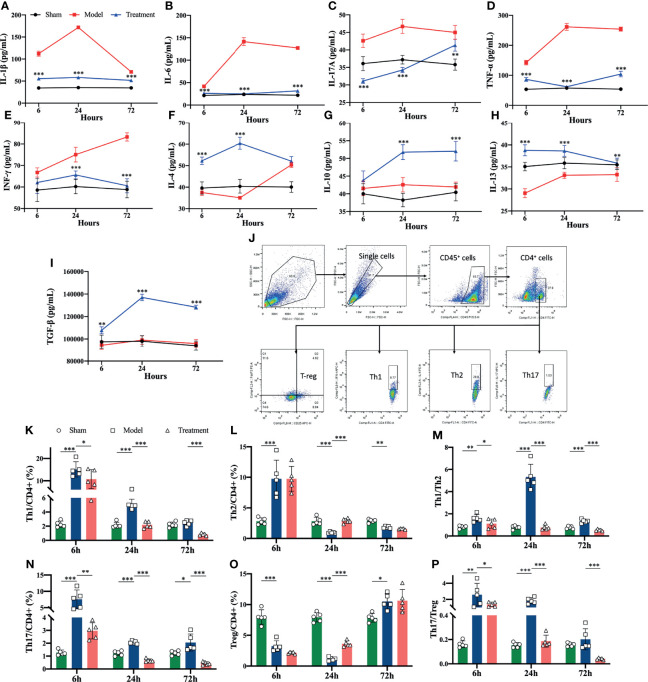
Levels of inflammatory cytokines and proportions of Th cell subpopulations in the spleen suspensions of rats in each group. **(A-I)** Levels of inflammatory cytokines in the spleen suspensions of rats in each group (compared with the model group); **(J)** circle-gating strategy for flow cytometry analysis of the proportion of splenic Th cells; **(K, L, N, O)** flow analysis of the proportion of Th1, Th2, Th17, and Treg cells in the spleen as a percentage of CD4^+^ T cells; **(M)** the ratio of Th1/Th2 in the spleen of rats in each group; **(P)** the ratio of The ratio of Th17/Treg in spleen of each group. n=5, *p<0.05, **p<0.01, ***p<0.001.

Flow cytometry revealed that the levels of pro-inflammatory cytokines were significantly higher in the spleens of aged sepsis model rats than the sham-operated rats; after MSC treatment, the levels of pro-inflammatory cytokines were reduced and the levels of anti-inflammatory cytokines were greatly increased among the treated rats (**Figures 4A–I**). The proportions of Th1 and Th17 CD4^+^ T cells in the spleen of aged sepsis rats were markedly increased at the early onset and gradually decreased thereafter; correspondingly the proportions of Th1 and Th17 cells were reduced after MSCs treatment, and the ratios of Th1/Th2 and Th17/Treg cells were also decreased notably(**Figures 4J–P**).

### Reduction of Apoptosis in the Spleen and Regulation STAT Signaling Activation Levels of MSCs Treatment in Aged Sepsis Rats

**Figure 5:**
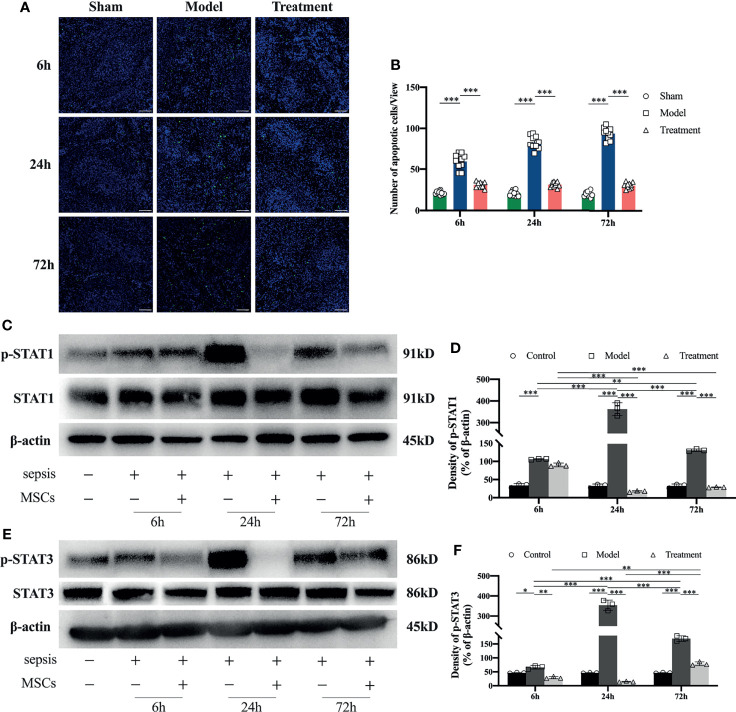
Tunel staining and STAT1 and STAT3 expression levels in the spleen of rats in each group. **(A)** Apoptosis of spleen lymphocytes at 6h, 24h and 72h in each group of rats (n=5, scale bar=100mm); **(B)** number of apoptotic cells per field of view in each group of rats; **(C, D)** expression levels and semi-quantitative analysis of STAT1 and p-STAT1 in spleen tissue of each group of rats (n=3); **(E, F)** expression levels and semi-quantitative analysis of STAT3 and p-STAT3 in spleen tissue of each group of rats (n=3). *p<0.05, **p<0.01, ***p<0.001.

Compared with the sham-operated group, the number of apoptotic cells in the spleen of rats in the control-treated group was obviously increased; a large number of apoptotic cells began to appear from the early onset and persisted its appearance. While in the MSCs-treated group, the number of apoptotic cells in the spleen of MSC-treated rats was remarkably lower than that of the control-treated group and similar to the sham-operated group with no significant apoptosis observed (**Figures 5A, B**). Through the tests by western blot of STAT1 and STAT3, it revealed that the phosphorylation levels of STAT1 and STAT3 in the spleens of aged sepsis model rats were greatly increased at all time points compared with those in the spleens of normal rats, and maximal phosphorylation was observed at 24 h (**Figures 5C–F**). The phosphorylation levels of STAT1 and STAT3 in the MSCs-treated rats were outstandingly lower than those in the control-treated rats at the same time points.

### Amelioration of LPS-Induced Inflammatory Responses in Lymphocytes of MSCs Treatment

**Figure 6:**
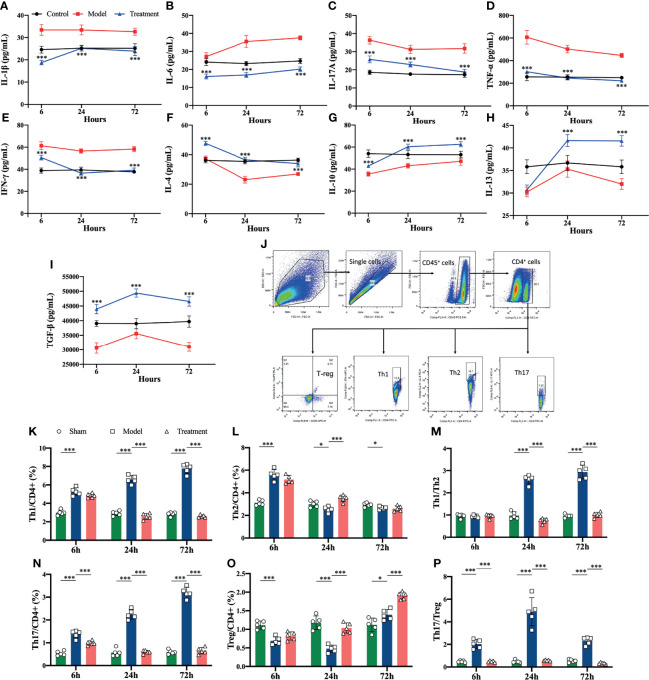
Levels of inflammatory cytokines in cell supernatants and the proportion of Th cell subpopulations in lymphocytes in each group of *in vitro* experiments. Lymphocytes isolated from the spleen were co-cultured with MSCs at a ratio of 10:1, and the lymphocytes and supernatants were analyzed at 6h, 24h and 72h, respectively. **(A–I)** Levels of inflammatory cytokines in cell supernatants of each group (*compared with the model group); **(J)** circle gating strategy for flow cytometry analysis of the proportion of Th cells in lymphocytes of each group; **(K, L, N, O)** flow analysis of the proportion of Th1, Th2, Th17, and Treg cells in lymphocytes of each group as a percentage of CD4^+^ T cells; **(M)** ratio of Th1/Th2 in lymphocytes of each experimental group; **(P)** ratio of Th17/Treg in lymphocytes of each experimental group. n=5, *p<0.05, ***p<0.001.

Compared to blank controls, LPS-stimulated lymphocytes produced a large number of cytokines, of which the levels of pro-inflammatory cytokines were evidently elevated. In contrast, it was observed that in the supernate of LPS-stimulated Lymphocytes co-cultured with MSCs, the levels of pro-inflammatory cytokines were reduced and the levels of anti-inflammatory cytokines were significantly boosted (**Figures 6A–I**). In addition, the flow cytometry detection presented that the proportions of Th1 and Th2 cells were obviously higher in LPS-stimulated lymphocytes and the ratios of Th1/Th2 and Th17/Treg cells were also evidently higher; while the ratios of Th1/Th2 and Th17/Treg cells in the co-culture group was notably lower than that in the LPS-stimulated group, which was consistent with the results of *in vivo* experiments (**Figures 6J–P**).

### Inhibitors Validation of the Role of the JAK-STAT Signaling Pathway

**Figure 7:**
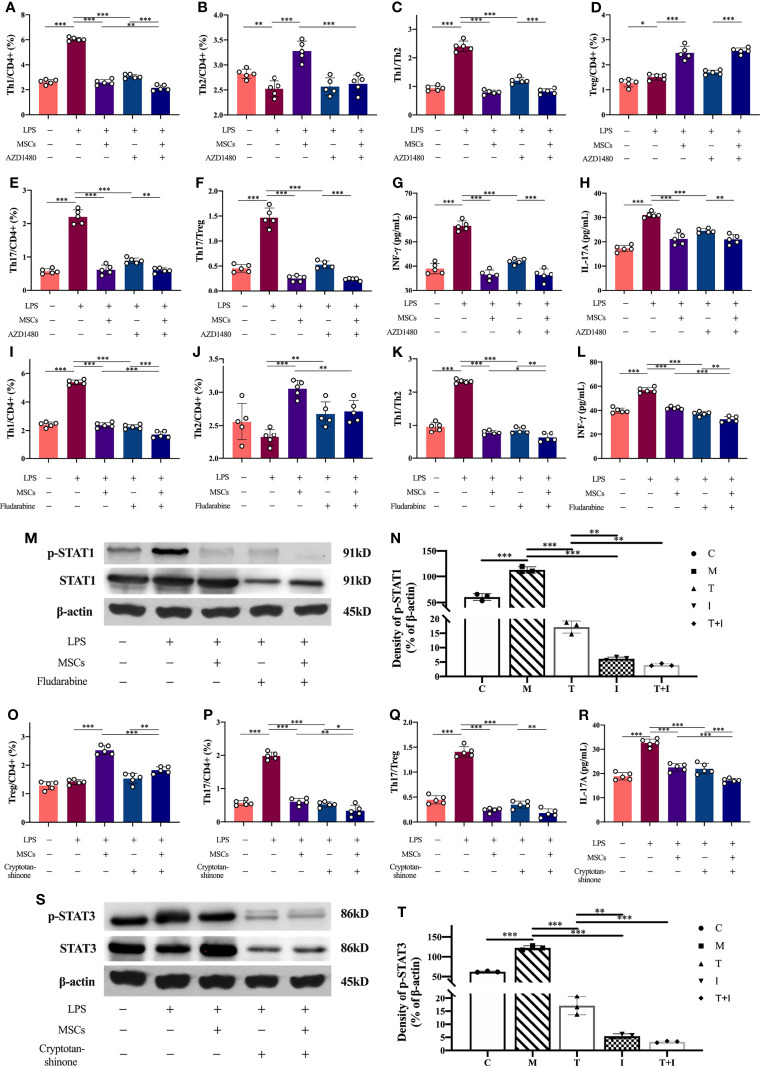
Levels of inflammatory factors in cell supernatants and the proportion of Th cells in lymphocytes and the expression levels of STAT1 and STAT3 in each group after the use of inhibitors. Lymphocytes isolated from rat spleen were co-cultured with MSCs at a ratio of 10:1, and the inhibitors used and the co-culture time was 24hours. The concentrations of the inhibitors used in the experiment were AZD1480 (5μM), fludarabine (100μM) and cryptotanshinone (50μM). **(A–F)** Proportions of Th1, Th2, Th17 and Treg cells in CD4+ T cells and the ratios of Th1/Th2 and Th17/Treg in each experimental group after the use of JAK inhibitors (n=5); **(G)** levels of IFN-γ in the supernatant of each experimental group after the use of JAK inhibitors (n=5); **(H)** levels of IL-17A in the supernatant of each experimental group after the use of JAK inhibitors (n=5); **(I–K)** Proportions of Th1 and Th2 cells in CD4+ T cells and Th1/Th2 ratio in each experimental group after using STAT1 inhibitor (n=5); **(L)** levels of IFN-γ in cell supernatants in each experimental group after using STAT1 inhibitor (n=5); **(M, N)** expression levels of STAT1 and p-STAT1 in lymphocytes in each experimental group and the results of semi-quantitative analysis (n=3); **(O–Q)** Proportions of Th17 and Treg cells to CD4+ T cells and Th17/Treg ratio in each experimental group after using STAT3 inhibitor (n=5); **(R)** Levels of IL-17A in cell supernatants of each experimental group after using STAT3 inhibitor (n=5); **(S, T)** Expression levels of STAT3 and p-STAT3 in lymphocytes of each experimental group and the results of semi-quantitative analysis (n=3). *p<0.05, **p<0.01, ***p<0.001; C, control group; M, model group; T, treatment group; I, inhibitor group; T+I, inhibitor+MSCs group.

The administration of JAK inhibitor (AZD1480) resulted in a distinct decrease in the ratios of Th1 and Th17 in lymphocytes compared to the LPS-stimulated samples, similarly the ratios of Th1/Th2 and Th17/Treg were reduced. The levels of IFN-γ (Th1-specific cytokine) and IL-17A (Th17-specific cytokine) in the supernate of the inhibitor-treated cells were also decreased. Furthermore, the ratios of Th1 and Th17 and the levels of their specific cytokines were reduced in the MSCs co-cultured group after the addition of JAK inhibitors, compared to the inhibitor-treated cells, and the difference was statistically significant (**Figures 7A–H**). The proportion of Th1 cells and the levels of IFN-γ in lymphocytes were evidently reduced after the administration of STAT1 inhibitor, that were consistent with the changes in the co-cultured group. STAT1 inhibitor would reduce the levels of STAT1 in lymphocytes, and the levels of p-STAT1 were markedly lower than those in the LPS-stimulated cells (**Figures 7I–N**). Similarly, the proportion of Th17 in lymphocytes and the level of IL-17A were greatly reduced after using the of STAT3 inhibitor, that were consistent with the changes in the co-cultured group; and the level of STAT3 in lymphocytes was reduced after the addition of the inhibitor, meanwhile the levels of p-STAT1 were evidently lower than those in the LPS-stimulated cells (**Figures 7O–T**).

## Discussion

In this study, we established a sepsis model using naturally aged SD rats to investigate the role and possible mechanisms of MSCs in treating sepsis in the elderly, regulating the immune inflammatory status and protecting organ functions. The results indicated that MSCs derived from the umbilical cord could improve the overall status and 72 h survival of aged rats with sepsis and protect vital organ functions and ameliorate histopathological damages caused by sepsis. The applied *in vivo* and *in vitro* experiments confirmed that MSCs could inhibit the systemic inflammatory response, regulate the Th1/Th2 and Th17/Treg ratios and inflammatory cytokine levels in aged rats with sepsis, and the modulation of the JAK-STAT signaling pathway may be one of the mechanisms of action.

Sepsis is a life-threatening organ dysfunction caused by a dysregulated host response to infection (1), and the intense early inflammatory response would cause massive depletion of immune cells and immune factors, which is an important cause of immune suppression in the later stages of sepsis (27). The presence of immune decline in the elderly itself, to some degree, would affect the function of all types of immune cells of the innate and adaptive immune systems, especially the progressive decline of the function of CD8^+^ T cells and CD4^+^ T cells, which would lead to a higher susceptibility to sepsis and mortality among the elderly (28). Currently, several studies have shown that MSCs can reduce the mortality and the degree of organ damage in sepsis model animals (29, 30), and the results of the latest preclinical meta-analysis have shown that MSCs treatment can outstandingly reduce the mortality rate in sepsis animals. Current experimental results could support a potential therapeutic effect of MSCs in clinical trials (31). To date, all three completed phase I clinical trials have confirmed the safety of MSCs applied to the treatment of patients with sepsis or endotoxemia (32–34). However, there is still no published study on the application of MSCs in aged animals with sepsis or in elderly sepsis patients. We injected MSCs of umbilical cord origin into naturally aged rats with sepsis and found that the mental and feeding status of the treated sepsis model rats were improved, and that the 24 h and 72 h mortality rates were distinctly reduced. Survival analysis revealed that the death of rats in the treated group was concentrated within 24 h after the completion of the CLP operation, which might be due to the organ dysfunction or even failure caused by the outbreak of inflammatory response. In contrast, the death of rats in the MSC-treatment group occurred more randomly and the survival time was longer compared with that of the control-treated group, which indicated the important protective role of MSCs at the early stage of the disease onset.

Current studies confirm that the application of MSCs for sepsis not only improves the survival rate of experimental animals but also improves several vital organ functions. Clinically, patients with sepsis have a high morbidity and mortality rate (35, 36), and in animal experiments, CLP sepsis animals had a 7-day mortality rate of 70% (37) and a 28-day mortality rate of 80% (11). After treatment with MSCs, the survival rates were both remarkably improved, critical organ functions in lung, liver, and spleen, etc. were effectively protected, and pathological damages to vital organs were obviously reduced (38). However, some researchers found that MSCs did not improve mortality or systemic inflammation and stress response in pigs with sepsis (39). In our study, we found that MSCs could significantly improve the pathological damage to vital organs, reduce the serum levels of ALT, AST, BUN, Cr and LDH and increase the level of ALP in aged sepsis model rats to a certain degree. These results indicated that MSCs possess good therapeutic effects in the aged sepsis model rats, which can effectively reduce their organ damage and protect organ functions, and further improved the survival rate of aged sepsis model rats. Through histological staining, we found that local infiltration of inflammatory cells was reduced after MSCs treatment. Accordingly, we speculated that the amelioration of the inflammatory factor storm in the early stage of sepsis might be an important mechanism by which MSCs exert their protective effects.

The cytokine storm is an excessive immune response induced by various stimuli, which is the most important pathophysiological feature of early sepsis and the main cause of multi-organ dysfunction and long-term immune suppression (40). Immune cells play a crucial role in the initiation phase of the cytokine storm (41). When the organism is stimulated by pathogenic microorganisms, the activated CD4^+^ T cells would be differentiated into different various subpopulations to play diverse roles; they are mainly divided into pro-inflammatory cell subpopulations represented by Th1 and Th17 and anti-inflammatory cell subpopulations represented by Treg and Th2 (42). Sepsis is a dysregulation of the pro-inflammatory/anti-inflammatory response caused by severe infection, as evidenced by an imbalance in the ratios of Th1/Th2 and Th17/Treg cells (43–45). In the early stage of infection, a variety of inflammatory cells are recruited and activated to release large amounts of inflammatory cytokines and chemokines, such as TNF-α and IL-1β, which are rapidly secreted and reach to the peak within a few hours. Under normal conditions, the body regulates the degree of inflammatory response by secreting anti-inflammatory factors to remove harmful substances and maintain intracellular homeostasis (46, 47). However, when this balance is disrupted, early reactive cytokines will trigger a cascade of additional cytokines further, which will lead to the activation and release of a large number of inflammatory factors, including IL-6, IL-12, and macrophage inflammatory protein (MCP)-1α, and induces an uncontrolled systemic inflammatory response (48, 49). MSCs have been found from other studies to reduce the levels of inflammatory factors in the serum of adult sepsis animal studies (31, 50), and this has been confirmed in aged sepsis model animals here. In our study, we found that the ratios of Th1/Th2 and Th17/Treg cells were significantly elevated in the peripheral blood of aged sepsis model rats, which indicated that helper T-cell subsets has played a certain role in the systemic inflammatory response. We also found that the ratios of Th1/Th2 and Th17/Treg cells were evidently reduced after treatment with MSCs and approached to the levels compared to the sham-operated group. Furthermore, cytokines associated with helper T cells, such as IFN-γ, IL-17, IL-4 and IL-10, presented the same trend as the change in the ratio of immune cells, which also confirmed that MSCs regulated the secretory function of Th cells and kept the excessive inflammatory response under control. In addition, MSCs were able to reduce the levels of pro-inflammatory cytokines such as IL-1β, IL-6 and TNF-α and elevate the levels of anti-inflammatory cytokines such as IL-10 and TGF-β in the circulation of aged sepsis model rats. This prevented the initiation and development of cytokine storms to a certain extent. Based on the *in vivo* experiments, we established an *in vitro* sepsis model by adding LPS to rat lymphocytes cultured *in vitro*. The results showed that, compared with the LPS-stimulated sample, the cells co-cultured with MSCs produced prominently less pro-inflammatory factors and more anti-inflammatory factors at all time points, and that the ratios of Th1/Th2 and Th17/Treg cells were decreased in the culture medium. From these results, we can conclude that MSCs can regulate the inflammatory response *in vitro* and *in vivo* by regulating the number and function of Th cell subpopulations.

The spleen, as the largest peripheral immune organ, accumulates large number of lymphocytes and is an important site for the specific immune response of the organism. Sepsis induces increased apoptosis of B lymphocytes and effector T cells, which further results in immune paralysis of the organism against subsequent infections. Current studies have verified that sepsis causes apoptosis of a large number of lymphocytes in the spleen, and the number of apoptotic splenocytes and lymphocytes is closely related to the severity of inflammation and patient prognosis (51). In patients with sepsis, the rate of lymphocyte apoptosis was obviously higher among those who died within 28 days after disease onset than those who were alive on day 28 (52). Using TUNEL staining of spleen tissues of aged sepsis model rats in each experimental group, we found that the proportion of apoptotic cells was significantly higher in the spleen of aged sepsis model rats, and that the number of apoptotic cells was distinctly reduced after treatment with MSCs. This indicated that MSCs can inhibit lymphocyte apoptosis induced by sepsis and suggested that MSCs had a positive effect on preventing immune dysfunction and long-term immunosuppression. To further investigate the source of changes on Th cells and inflammatory factors in the circulation, we examined the levels of inflammatory factors and the ratios of Th cell subpopulations in the spleen and found that the overall trends of changes in inflammatory factors and Th cell subpopulations in the spleen of rats in each group were consistent with those in peripheral blood. This indicates that MSCs can regulate the ratio of Th cells in the spleen and maintain the Th1/Th2 and Th17/Treg balance, which in turn regulates the levels of inflammation-related cytokines and controls the inflammatory response. After treatment with MSCs, the activation of Th1 and Th17 cells in the spleen of aged sepsis model rats was reduced, and the number of activated cells released into the peripheral blood was correspondingly reduced; and thus regulated the inflammatory response and exerted a protective effect on all organs of the body.

The JAK-STAT pathway is capable of mediating cell proliferation and apoptosis (53), which is thought to be involved in sepsis-induced multi-organ dysfunction (12).The JAK-STAT signaling pathway is activated by both pro-inflammatory cytokines (IFN-γ, IL-12, and IL-27) and anti-inflammatory cytokines (IL-4, IL-10, and IL-13), which indicates that individual STAT family members would deferentially regulate the balance of Th cell subsets (54). In the helper T-cell subpopulation, Th1 cell differentiation is mainly regulated by STAT1 and STAT4 (55), while STAT6 and STAT3 regulate the differentiation of the immunoregulatory Th2 and Th17 cells, respectively (56). One study showed that STAT1 knock-out mice were distinctly resistant to LPS-induced endotoxemia (57) and CLP-induced septic shock (58). This was attributed to the altered balance of the Th1/Th2 immune response, which resulted in the prominent reduced of organ damage in the liver and kidney. Another study showed that JAK2 inhibitors improved the survival rate after CLP operation in mice and rats by reducing the expression of pro-inflammatory mediators such as TNF-α, IL-6 and high mobility group protein box-1 (HMGB-1) (59). To explore the molecular mechanism of Th cell regulation by MSCs, we examined the relevant STAT signaling pathways in the spleen of aged sepsis model rats. The results indicated that the phosphorylation levels of STAT1 and STAT3 were significantly increased in the spleen of sepsis model rats, the phosphorylation levels of both signaling pathways were remarkably reduced after MSCs treatment, and the expression levels of STAT1 and STAT3 were similar to those of normal rats. In the *in vitro* experiments, we administered inhibitors of the JAK, STAT1 and STAT3 signaling pathways to the LPS-stimulated cells and LPS-stimulated cells co-cultured with MSCs. The results showed that the proportion of Th cell subpopulations in the LPS-stimulated sample changed after treatment with individual inhibitor, and the overall trend of the changes was consistent with that in the LPS+MSC cells. This indicated that MSCs and JAK-STAT inhibitors exerted the same effect, while the results of LPS+MSCs treated with inhibitors showed that MSCs and inhibitors could function synergistically to some extent. In addition, we evaluated the levels of IFN-γ and IL-17, which represent Th1 and Th17 cells, respectively, in each sample. It was found that JAK inhibitors were able to reduce the levels of both IFN-γ and IL-17, while STAT1 inhibitor reduced the levels of IFN-γ and STAT3 inhibitor reduced the levels of IL-17. The above results confirm that MSCs regulate the quantity and function of the relevant Th cell subpopulations by inhibiting the JAK-STAT signaling pathway to control the inflammatory response. If this is the case, can JAK inhibitors be used directly to treat sepsis? The answer is uncertain. The MSCs used in this study have more diverse regulatory functions and effects than the single effect of JAK-STAT pathway inhibitors. The mechanisms of immunomodulatory effects of MSCs are complex which would involve the synthesis and secretion of multiple mediators, direct interaction with target cells, and regulation by certain antigen-presenting cells. In MSCs, no single pathway controls the entire process (60), and their regulatory effects are influenced by the microenvironment and immune status. Thus, we confirmed that MSCs could protect aged sepsis model rats through the regulation of the JAK-STAT signaling pathway; we also acknowledge that this is only the tip of the iceberg of the mechanism of action of MSCs, and that more complex mechanisms remain to be investigated in depth.

Although our study confirmed the protective effect of MSCs in aged sepsis model rats, there are still some limitations: our understanding of the molecular mechanism of Th cell regulation by MSCs is incomplete; we also have not clarified whether the immune function of aged sepsis model rats is different or similar to that of adult sepsis model rats. We will continue to investigate related issues in depth in our subsequent studies.

In conclusion, this study confirms that umbilical cord MSCs can improve the survival rate of aged sepsis rats. The mechanism may be through the regulation of helper T cell subsets and inflammatory factor storm, thus ameliorating immune dysfunction and tissue and organ damage. Inhibition of the JAK-STAT signaling pathway may be one of the molecular mechanisms of UC-MSCs in the treatment of sepsis. UC-MSCs could be a potential treatment for sepsis in old age and deserve further investigation.

## Data Availability Statement

The raw data supporting the conclusions of this article will be made available by the authors, without undue reservation.

## Ethics Statement

The animal study was reviewed and approved by Ethics Committee of the Chinese PLA General Hospital.

## Author Contributions

LW performed experiments, analyzed data, and wrote the manuscript. ZD and YS performed experiments and analyzed data. YL, YZ, and YYL performed experiments. MY and RY analyzed data. FZ revised the manuscript. ZQ guided the experimental design. HK designed and guided the experiments and revised the manuscript. All authors contributed to the article and approved the submitted version.

## Conflict of Interest

The authors declare that the research was conducted in the absence of any commercial or financial relationships that could be construed as a potential conflict of interest.

## Publisher’s Note

All claims expressed in this article are solely those of the authors and do not necessarily represent those of their affiliated organizations, or those of the publisher, the editors and the reviewers. Any product that may be evaluated in this article, or claim that may be made by its manufacturer, is not guaranteed or endorsed by the publisher.
