# Supplementary material for: The Study on the Regulation of Th Cells by Mesenchymal Stem Cells Through the JAK-STAT Signaling Pathway to Protect Naturally Aged Sepsis Model Rats
Source: Front Immunol. 2022 Feb 7;13:820685. doi: 10.3389/fimmu.2022.820685 (PMC8858840; doi:10.3389/fimmu.2022.820685)
Supplement: Supplementary file 1 [file DataSheet_1.docx]

Supplementary Material

## Supplementary Figures


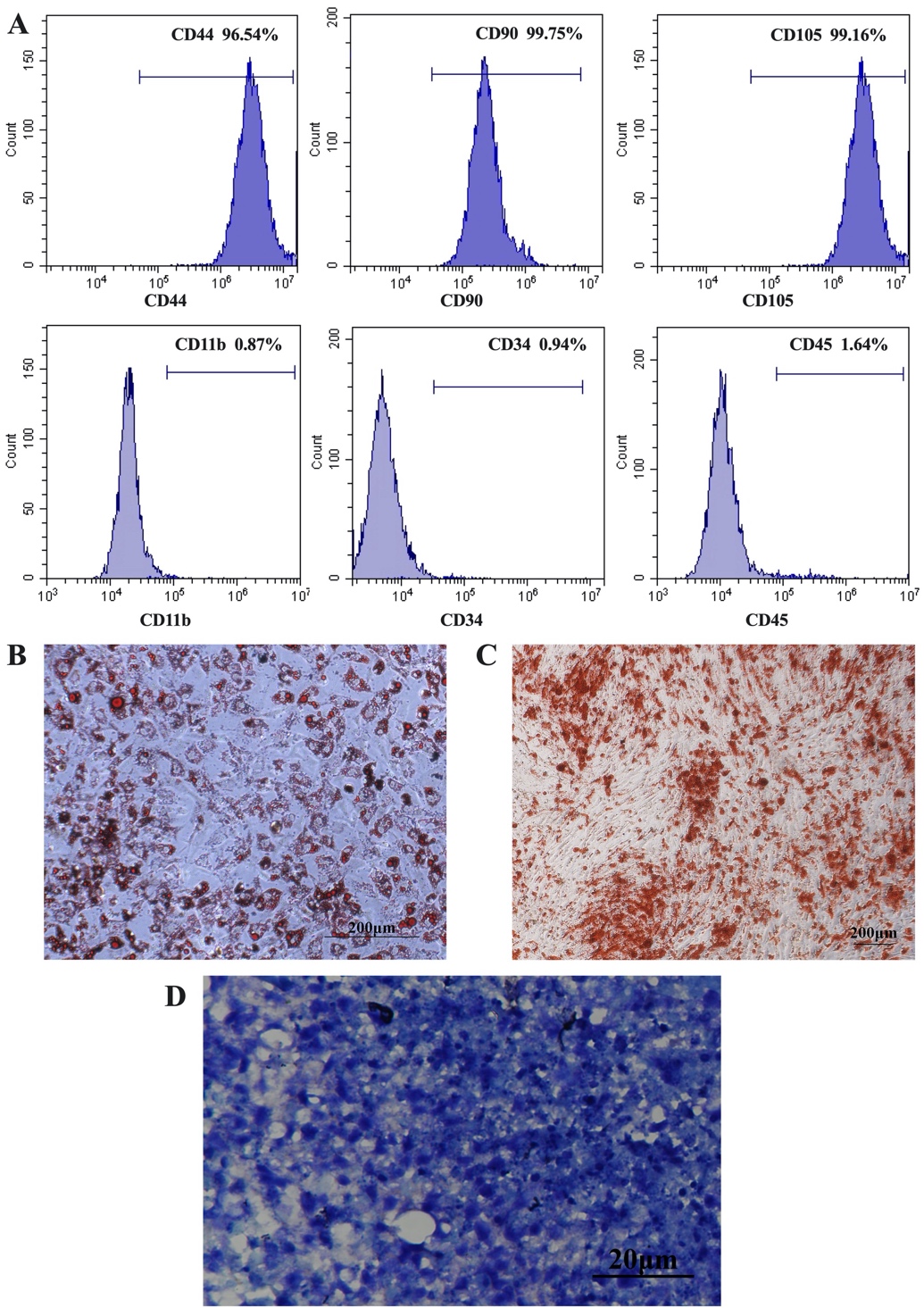


**Supplementary Figure 1.** Characteristics of umbilical cord-derived mesenchymal stem cells (UC-MSCs). The UC-MSCs were identified by their phenotypes and the potential for differentiating into adipocytes, osteoblasts and chondrocyte. (A) For immunological phenotypes, the isolated and cultured cells were positive for CD44, CD90 and CD105, and negative for CD11b, CD34 and CD45. (B) Differentiation of cells into adipocytes was confirmed by oil red O staining. Scale bar, 200μm. (C) Differentiation of cells into osteoblasts was confirmed by alizarin red staining. Scale bar, 200μm. (D) Differentiation of cells into chondrocyte was confirmed by toloniumchloride staining. Scale bar, 20μm.
